# EUPopLink Country report - Luxembourg

**DOI:** 10.12688/openreseurope.23514.1

**Published:** 2026-05-05

**Authors:** Lisa Verhasselt

**Affiliations:** 1 Luxembourg Institute of Socio-Economic Research, Esch-sur-Alzette, Luxembourg District, Luxembourg

**Keywords:** populism, euroscepticism, Luxembourg

## Abstract

This report examines the dynamics of populism and Euroscepticism in Luxembourg, focusing on the Alternative Democratic Reform Party (ADR) as the country’s principal right-wing populist actor. Luxembourg exhibits a stable pro-European consensus shaped by its small size, economic reliance on EU integration, and absence of organized anti-EU interest groups. Public attitudes reflect this elite-driven pro-EU stance, with high levels of attachment and optimism toward the EU. The ADR, originally a single-issue pension reform party, has evolved into a soft right-wing populist force, emphasizing national sovereignty, cultural preservation, and pragmatic critiques of EU policies rather than advocating withdrawal. Its discourse combines vertical exclusion (people vs. elites) and horizontal exclusion (people vs. migrants), while adapting to Luxembourg’s socio-economic context, particularly its highly integrated labor market and multicultural population. Mainstream parties respond adversarially to ADR initiatives when perceived as crossing political or ethical boundaries, yet occasional rhetorical adjustments suggest subtle accommodation of public concerns. Luxembourg thus illustrates a model of ‘chameleonic populism,’ where populist and Eurosceptic ideas are moderated and contextually tailored, highlighting the influence of structural and socio-political conditions on the expression of exclusionary politics in small, integrated states.

## Introduction

As a founding member of the EU and host to several major EU institutions, Luxembourg has a political landscape characterized by a strong and consistent pro-European consensus. This consensus is likely influenced by two factors. First, the country’s small size and its economic dependence on European integration, including foreign and cross-border labour. Second, Luxembourg lacks organized interest groups, business stakeholders, or political parties advocating for withdrawal from the EU ( Besch & Lessing, 2016). As a result, “strong Euroscepticism is basically non-existent in public discourse” (ibid, p. 12).

Public attitudes toward the EU in Luxembourg largely mirror those of the political elite, reflecting a consistently high level of pro-European sentiment. According to the Spring 2025 Eurobarometer, 57% of Luxembourgish respondents view the EU positively (compared to the EU average of 43%), 66% are optimistic about its future (EU average: 62%), and 81% feel attached to the EU (EU average: 63%). These figures indicate a limited public demand for a strong Eurosceptic party. While high levels of support for the EU may appear expected given Luxembourg’s deep integration into the Union, the country’s case is particularly striking in light of its socio-economic profile – one that encompasses themes often exploited by right-wing populists, such as globalization and migration. Nonetheless, Luxembourg presents a notable counterexample: it ranks among the countries with the highest GDP per capita globally, maintains a labour market heavily dependent on foreign workers, and offers many native Luxembourgers stable, well-paid employment in the public sector ( de Jonge, 2021). Furthermore, despite having one of the highest proportions of immigrants relative to its population in Europe, Luxembourg is frequently cited as a successful case of integration ( Fetzer, 2011), further reinforcing public support for the EU’s core values.

Ultimately, Euroscepticism has remained a marginal force in both party politics and public discourse, with Luxembourg not typically being associated with populism ( Carls, 2024, 2021; de Jonge, 2021; Fetzer, 2011).

## Populist political actors

The primary vehicle for right-wing populist discourse in Luxembourg is the Alternative Democratic Reform Party (Alternativ Demokratesch Reformspartei, ADR). Founded in 1987 as a single-issue movement advocating pension reform, the ADR gradually broadened its platform, renaming itself in 1992 to highlight democracy and pension justice. Its electoral peak came in 1999 with 11.3% of the national vote. Following pension reforms in 1998 and 2002, which reduced the relevance of its core issue ( Schulze, 2006), the party rebranded in 2006 to reflect wider political ambitions. The ADR regained visibility in 2015 by leading the successful opposition to a referendum on extending voting rights to non-citizen residents. As of the 2023 general election, it holds five seats in the 60-member Chamber of Deputies and, for the first time, gained a seat in the European Parliament in 2024.

The ADR was not conceived as a right-wing party, but only shifted in that direction in the early 2000s. Accordingly, scholars are divided on labelling it as right-wing populist: some support the label ( Carls, 2024, 2021; de Jonge, 2021; Zulianello, 2020; Blau, 2005), while others reject it ( Luxemburger Wort, 2017; Poirier, 2012). This ambiguity reflects Luxembourg’s affluent, consensus-based society, which curbs hard-line populism. Consequently, the ADR is best seen as a soft version of right-wing populism ( Blau, 2005), moderating its rhetoric to appeal to a centrist electorate ( Carls, 2021). Its ideology blends national conservatism with classical liberalism, stressing national sovereignty, cultural preservation, and direct democracy. The ADR is Luxembourg’s most immigration-sceptical party ( Fetzer, 2011, p. 15), opposing foreign voting rights and favouring restrictive citizenship laws. Socially conservative on gender issues, it resists gender ideology and expanded transgender rights but supports liberal stances on topics such as prostitution ( Carls, 2021, 2024). It also criticizes state overreach, particularly evident in its opposition to COVID-19 restrictions at national and EU levels.

The ADR joined the Alliance of Conservatives and Reformists in Europe (ECR) in 2010, drawing occasional accusations of far-right sympathies – claims the party strongly rejects. In 2023, Fernand Kartheiser, then MP and now MEP, asserted: “There is no extreme right politically represented in Luxembourg. All these extremist tendencies are completely alien to our thinking.” Yet historian Blau has challenged this, arguing that the ADR’s emphasis on national identity, Euroscepticism, moral decline, and immigration reflects core themes of the European far right ( RTL Today, 2023). Similarly, de Jonge ( Journal.lu, 2023; RTL Today, 2024b) observes a clear rightward shift, particularly in the party’s increased focus on migration, gender, and climate – issues commonly associated with far-right agendas – though she refrains from labelling the ADR as extremist. The likely departure of former president and founding member Jean Mehlen, citing “ideological differences,” further underscores concerns about the party’s radicalisation ( RTL Today, 2025a).

## Positions on ‘Europe’ and the Populism-Euroscepticism nexus

Luxembourg’s political landscape is anchored in a stable pro-European consensus, with major parties like the Christian Social People’s Party (CSV), the Democratic Party (DP), the Luxembourg Socialist Workers’ Party (LSAP), and the Greens (Déi Gréng) supporting EU integration. In this landscape, Euroscepticism remains marginal. While parties such as The Conservatives (right-wing), The Left (left-wing), and the Luxembourg Communist Party (left-wing) express varying Euroscepticism, the ADR stands out as the leading right-wing populist Eurosceptic force. However, compared to similar European movements, it defends a relatively soft variety of Euroscepticism – often earning it the label of ‘Eurocritical’ rather than outright Eurosceptic.

Since moving beyond pension reform, the ADR has adopted a soft Eurosceptic stance. It has opposed treaties like the EU Constitution and the Lisbon Treaty, yet continues to support the EU, the euro, and the single market. The party advocates for a ‘Europe of Nations’ rooted in intergovernmental cooperation and routinely criticizes perceived EU overreach, particularly in areas such as migration, identity, and sovereignty. Luxembourg’s small size heightens its concerns about marginalization within EU decision-making, though the ADR does not advocate withdrawal. As party president Alexandra Schoos has affirmed, “Luxembourg needs Europe and the European Union ( Le Quotidien, 2024).” Symbolic efforts, such as promoting Luxembourgish as an official EU language, highlight its desire to preserve national identity within the broader European project.

The ADR has reaffirmed its long-standing concerns and critiques about sovereignty, centralisation, and elite governance during recent crises. During the Brexit debate, the party acknowledged Luxembourg’s economic dependence on the EU, adopting a pragmatic pro-EU stance while framing the moment as an opportunity to advocate for the repatriation of certain competences to the national level ( Högenauer, 2021). In the COVID-19 pandemic, it criticized restrictive measures, opaque decision-making, and unilateral border closures as emblematic of supranational overreach and a failure to protect smaller states ( Lamour & Carls, 2022). In response to the war in Ukraine ^i^, the ADR condemned Russia’s invasion but criticized EU sanctions and defence federalisation, calling instead for a sovereignty-first and economically cautious approach. Across these crises, the party has not fundamentally shifted but rather deepened its longstanding positions, reinforcing its image as a critical reformer rather than an outright rejectionist.

Notably, the ADR adapts its populism to Luxembourg’s specific political and cultural context, aligning itself with broader meta-populist themes while maintaining local resonance. It engages in both vertical exclusion (people versus elites) and horizontal exclusion (people versus migrants), dynamics clearly evident in the 2024 European Parliament campaign. At the EU level, the party targeted Brussels elites, the ECB, and technocrats, while also criticising ‘outsiders’ such as third-world immigrants and green-left ideologues – narratives echoed in national politics ( Carls, 2024).

The ADR portrays Luxembourg as a multicultural space rather than a multicultural nation, welcoming foreigners for economic purposes while denying them inclusion in the national identity. This positioning complicates classic populist dichotomies. Rather than defending the marginalized, the ADR advances a ‘populism from above’, aligning with the relatively privileged native population – particularly those in public administration and education, sectors dominated by Luxembourgers and requiring fluency in Luxembourgish. Language, in this context, becomes both a tool of integration and exclusion. The ADR’s defence of Luxembourgish is not only cultural but also economic, protecting access to high-paying public sector jobs for native speakers. In this way, the party appeals to a conception of ‘the people’ that is atypical of right-wing populism ( Carls, 2021).

In sum, the ADR shares key characteristics with broader European right-wing populist movements, particularly its opposition to Islam, multiculturalism, and political correctness. Yet its engagement in a wider meta-populist discourse, coupled with its distinctive approach to multicultural integration, helps explain why it often resists easy classification. The party embodies what Taggart (2000, 2004) terms ‘chameleonic populism’ and exemplifies Carls’ (2021) notion of ‘right-wing populism à la Luxembourgeoise’: EU-critical without being anti-EU, culturally defensive yet economically pragmatic. Its brand of populism and Euroscepticism is shaped by Luxembourg’s unique socio-political context, forcing the ADR to balance national sovereignty with European integration in ways that reflect the country’s deep embeddedness in the EU. Ultimately, the ADR’s soft Euroscepticism sets it apart in a national political landscape where hard-line anti-European nationalism has gained little ground.

## Responses to Euroscepticism and consequences

In response to the rise of Eurosceptic and populist discourse – particularly that advanced by the ADR – Luxembourg’s pro-EU mainstream parties have mainly adopted an adversarial approach. Given the ADR’s relatively moderate and often subdued Euroscepticism, reactions to the party tend to be more pronounced at the European level than domestically.

At several critical junctures, Luxembourg’s mainstream parties have adopted an overtly adversarial stance, particularly when the ADR aligned itself with far-right groups in the EP. The ADR’s controversial decision to join the ECR was met with strong condemnation from MEPs representing the mainstream parties (DP, the LSAP, Déi Gréng, and CSV). MEP Charles Goerens (DP) described the ADR’s new allies as “not the best company,” while MEP Marc Angel (LSAP) warned that this marked “the first time that Luxembourg has members on the benches of the extreme right or the populist right,” arguing that such alliances aim to build a “weakened Europe.” MEP Tilly Metz (Déi Gréng) criticized the ADR’s populist approach, remarking that “it’s easy to run a campaign with populist slogans,” but stressing the lack of substantive policy proposals. MEP Christophe Hansen (CSV) underscored growing concerns by characterizing the ADR as part of “the far-right bloc” and called for greater cooperation among centrist parties to counter its influence ( Luxembourg Times, 2024b).

Recently, the ADR’s position on the war in Ukraine has sparked reactions from other political actors. MEP Isabel Wiseler-Lima (CSV) commented, “We observe that the ADR has become more radicalized. They express understanding for various Russian positions, which we find deeply concerning.” Hansen (CSV) draws parallels between the ADR and far-right parties elsewhere: “While the ADR may not have shifted dramatically to the far right on all issues, their stance on foreign policy – particularly regarding Ukraine – is unmistakably hard-line.” He notes that such parties often exploit public fears, especially around migration policies ( Virgule, 2024). Similarly, ADR MEP Kartheiser’s recent trip to Moscow and his subsequent expulsion from the ECR group triggered widespread criticism. Foreign Minister Xavier Bettel (DP) called the trip “extremely problematic and unhelpful ( Luxembourg Times, 2025b).” Metz (Déi Gréng) responded with sharp irony, stating, “Too extreme even for the extremists! That’s quite an achievement ( RTL Today, 2025c).”

The ADR’s emphasis on the Luxembourgish language, both at the EU and national levels, has also prompted other parties to adopt a distinctly adversarial stance. At the EU level, Kartheiser’s insistence on using Luxembourgish in the EP was met with ridicule. Wiseler-Lima (CSV) dismissed it as a “circus act,” while Angel (LSAP) described it as “a stunt” and a form of “populism,” stating, “We conduct politics for our language, not with it. That is what the far-right and the populists might do, but it’s not for me ( RTL Today, 2024c).” Domestically, mainstream parties have also pushed back against the ADR’s linguistic nationalism. MPs from across the political spectrum accused the party of distorting data and inflaming xenophobic sentiments. Culture Minister Eric Thill (DP) likened the ADR’s narrative to “a juggler throwing a single ball” – an illusion of complexity without real substance. Similarly, MP Liz Braz (LSAP) warned against instrumentalising language for political gain, asserting that it promotes “subliminal xenophobia in political discourse ( RTL Today, 2024a).”

Adversarial responses have also been evident in moments of heightened controversy. A notable example occurred in 2025, when ADR MP Tom Weidig liked a social media post advocating the “extermination of LGBTQ” individuals. The Chamber of Deputies responded with a rare unanimous resolution condemning all forms of hate speech ( RTL Today, 2025b). Another incident dates back to 2019, when a Facebook post by then-ADR politician Sylvie Mischel sparked outrage among political opponents. Then party leaders Djuna Bernard (Déi Gréng) and Franz Fayot (LSAP) compared the ADR to Germany’s right-wing populist Alternative for Germany (AfD), while Frank Engel (CSV) called on citizens to resist “sleeping fascism ( Reporter.lu, 2019).” These cases illustrate how verbal transgressions, including on social media, are met with sharp condemnation by Luxembourg’s mainstream parties.

Overall, mainstream parties adopt an adversarial posture when the ADR’s populist or Eurosceptic rhetoric crosses perceived red lines, employing terms like “right-wing extremism,” “senseless,” or “stunt” to delegitimize the party. In these moments, parties across the spectrum accuse the ADR of fostering a toxic and divisive political climate. At other times, however, their response is more dismissive, aimed at marginalizing the ADR’s populist tactics and curbing its influence.

However, as noted by de Jonge ( RTL today, 2024b), the growing influence of radical right-wing ideologies has prompted centrist parties to cautiously echo certain themes to maintain electoral competitiveness. For example, in 2024, MP Simone Beissel (DP) sparked controversy with remarks about beggars, particularly her references to the Sinti and Roma communities. Likewise, Marc Lies (CSV) faced backlash after sharing Facebook content that perpetuated negative stereotypes about refugees, unfairly portraying them as criminals. De Jonge situates these instances within a broader European pattern, where mainstream parties subtly adjust their rhetoric to address the concerns of right-leaning voters. While Luxembourg’s major parties have not openly embraced Euroscepticism, they have nevertheless adopted a cautiously accommodative rhetorical approach on select issues, indirectly responding to the ADR’s agenda.

Ultimately, the ADR’s discourse has not shifted Luxembourg’s fundamentally pro-European orientation. The prevailing pattern remains one of adversarial engagement, with mainstream actors committed to defending European integration while discrediting the ADR’s Eurosceptic populism and far-right expressions.

## Conclusion

While Luxembourg remains largely insulated from the surge of hard Euroscepticism and radical right-wing populism seen elsewhere in Europe, it is by no means immune to their influence. The ADR illustrates how these ideologies can be strategically adapted to a small, multilingual, and highly integrated state. Its brand of soft Euroscepticism and culturally defensive populism does not seek to dismantle the EU but rather challenges specific aspects of integration – particularly regarding migration, national sovereignty, and cultural identity –through a reformist rather than rejectionist lens.

Importantly, while no political party in Luxembourg currently advocates leaving the EU, and strong Euroscepticism remains unlikely to gain mass support ( de Jonge, 2021; Blau, 2005), this does not imply immunity to more subtle forms of right-wing or exclusionary thinking. As shown, such currents are becoming increasingly normalized – repackaged and marketed in ways that align with Luxembourgish sensibilities.

This case highlights the importance of political and structural context in shaping how populist and Eurosceptic sentiments manifest. In a country where pro-European sentiment is strong and no major actor advocates for EU withdrawal, the ADR has gained traction by repackaging nationalist and exclusionary ideas in ways that resonate locally. It tempers xenophobic rhetoric, endorses limited multiculturalism, and critiques EU policies in pragmatic, nationally focused terms. In doing so, it exemplifies ‘chameleonic populism’ ( Taggart, 2000, 2004): ideologically flexible, responsive to local conditions, and capable of working within the political mainstream even as it challenges aspects of it.

Luxembourg’s experience invites broader comparative reflection. In small, open states where economic prosperity relies on international labour flows and deep EU integration, populism may not disappear, but it does transform. Rather than outright radicalism, what emerges are moderated, locally adapted forms of right-wing populism that emphasize cultural protection and sovereignty while avoiding open hostility to immigration or European cooperation. These environments do not eliminate radical tendencies but instead reshape them, allowing exclusionary ideas to gain acceptance under the guise of cultural preservation and economic pragmatism. In this light, Luxembourg’s case underscores the need for more nuanced, context-sensitive approaches to the study of European populism – approaches that account for political geography, demographic complexity, and the varying degrees of structural embeddedness in the EU. It challenges large-state paradigms and broadens the analytical scope to include the unique pressures and adaptations found in small-state contexts.

In conclusion, political parties reflect the preferences of their electorates. In Luxembourg, there is little appetite for a party that is aggressively anti-immigration or openly anti-EU – largely because the country benefits profoundly from both. Yet the ADR demonstrates that even in such a context, populism can find fertile ground by adapting its discourse to the structures, values, and identity concerns of the national context. As such, Luxembourg stands as a distinctive and instructive case for understanding the flexible and multifaceted nature of populism and Euroscepticism in Europe today.

## Ethics and consent

Ethical approval and consent were not required.

## Data Availability

No data are associated with this article.

## Footnotes

^i^
However, some of the ADR’s leading figures have, at times, openly expressed support for more radical right-wing positions. This tendency is particularly evident in the context of the war in Ukraine, where Kartheiser’s rhetoric has sparked controversy. His framing of the Ukraine war as a U.S.-Russia proxy conflict and criticism of EU alignment with the Biden administration echo narratives associated with Donald Trump (two-thirds of ADR voters thought that the election of Donald Trump is a good thing) ( Luxembourg Times, 2024a). Kartheiser’s 2025 trip to Moscow – and his remark that he would “rather fly to Moscow than talk to the Brussels bubble” – marked a clear opposition to the EU. The fallout was swift: on 4 June 2025, Kartheiser was expelled from the ECR group – the ADR continues to stand with Kartheiser ( LuxembourgTimes, 2025a).
