# EUPopLink Country report - Luxembourg

**DOI:** 10.12688/openreseurope.23514.2

**Published:** 2026-08-06

**Authors:** Lisa Verhasselt

**Affiliations:** 1 Luxembourg Institute of Socio-Economic Research, Esch-sur-Alzette, Luxembourg District, Luxembourg

**Keywords:** populism, euroscepticism, Luxembourg

## Abstract

This report examines the dynamics of populism and Euroscepticism in Luxembourg, focusing on the Alternative Democratic Reform Party (ADR) as the country’s principal right-wing populist actor. Luxembourg exhibits a stable pro-European consensus shaped by its small size, economic reliance on EU integration, and absence of organized anti-EU interest groups. Public attitudes reflect this elite-driven pro-EU stance, with high levels of attachment and optimism toward the EU. The ADR, originally a single-issue pension reform party, has evolved into a soft right-wing populist force emphasizing pragmatic critiques of EU policies rather than advocating withdrawal. Its discourse combines vertical exclusion (people vs. elites) and horizontal exclusion (people vs. migrants), while adapting to Luxembourg’s socio-economic context, particularly its highly integrated labor market and multicultural population. Mainstream parties respond adversarially to ADR initiatives when perceived as crossing political or ethical boundaries, yet occasional rhetorical adjustments suggest subtle accommodation of public concerns. Luxembourg thus illustrates a model of ‘chameleonic populism,’ where populist and Eurosceptic ideas are moderated and contextually tailored, highlighting the influence of structural and socio-political conditions on the expression of exclusionary politics in small, integrated states.

## Introduction

As a founding member of the EU and host to several major EU institutions, Luxembourg is characterized by a broad and durable pro-European consensus. This consensus reflects the country’s small size and its economic dependence on European integration, including foreign and cross-border labour, as well as the absence of organized interest groups, business stakeholders, or major political parties advocating for withdrawal from the EU ( Besch & Lessing, 2016). Consequently, “strong Euroscepticism is basically non-existent in public discourse” (ibid, p. 12).

Public attitudes largely mirror this elite-level consensus. According to the Spring 2025 Eurobarometer, 57% of respondents in Luxembourg hold a positive image of the EU, compared with 43% across the EU; 66% are optimistic about its future, compared with 62%; and 81% feel attached to the EU, compared with 63% ( Eurobarometer, 2025). These levels of support substantially narrow the electoral space for hard Euroscepticism. Public demand is not fixed, however: parties can activate or amplify latent concerns through agenda-setting and issue framing. Luxembourg’s pro-European public opinion therefore constrains, but does not eliminate, opportunities for softer and more selective Eurocritical mobilization.

The case is nevertheless striking because several of Luxembourg’s socio-economic characteristics – globalization, high immigration, and intense labour mobility – are frequently mobilized by right-wing populists elsewhere. Luxembourg ranks among the countries with the highest GDP per capita globally, has one of Europe’s highest proportions of foreign residents and a labour market heavily dependent on foreign and cross-border workers. Of its residents, 12.9% originate from neighbouring countries, 20.9% from other EU member states, and 15.5% from outside the EU. Moreover, Luxembourg is among the OECD countries receiving the highest number of asylum applications per capita ( STATEC, 2021). Meanwhile, many native Luxembourgers occupy stable, well-paid public-sector positions for which Luxembourgish proficiency remains important ( de Jonge, 2021). Despite – or perhaps partly because of – these conditions, Luxembourg is frequently cited as a comparatively successful case of integration ( Fetzer, 2011).

Euroscepticism has thus remained marginal in both party politics and public discourse, and Luxembourg is not usually associated with populism ( Carls, 2021, 2024; de Jonge, 2021; Fetzer, 2011). It nevertheless constitutes a valuable least-likely case for examining the populism-Euroscepticism nexus. ^i^ It shows that these currents may emerge even under highly constraining conditions, although in moderated and locally adapted forms.

## Populist political actors

The principal actor combing right-wing politics with a recurring populist discourse in Luxembourg is the Alternative Democratic Reform Party (Alternativ Demokratesch Reformspartei, ADR). Founded in 1987 as a single-issue movement campaigning for pension reform, the ADR gradually broadened its programme and, in 1992, adopted a new name emphasizing democracy and pension justice. It reached its electoral peak in 1999, winning 11.3% of the national vote. After pension reforms in 1998 and 2002 reduced the salience of its founding issue ( Schulze, 2006), the party rebranded again in 2006 to reflect its wider political ambitions. It regained prominence in 2015 by leading the successful campaign against a referendum proposal to extend voting rights to non-citizen residents. In the 2023 general election, the ADR secured five of the 60 seats in the Chamber of Deputies, before winning its first seat in the European Parliament in 2024.

The ADR’s classification remains contested. Some scholars and expert-informed classifications describe it as populist, far right, or right-wing populist ( Carls, 2021, 2024; de Jonge, 2021; Zulianello, 2020; Blau, 2005), whereas others are more hesitant or reject these labels ( Luxemburger Wort, 2017; Poirier, 2012). This ambiguity partly reflects the party’s comparatively moderate rhetoric and adaptation to Luxembourg’s structural and socio-political constraints. A restrictive approach may regard its populism as too intermittent or muted, while a context-sensitive approach identifies a strategically adapted form of “right-wing populism à la luxembourgeoise” ( Carls, 2021).

Under the EUPopLink definition, populism requires both anti-elite claims and appeals to popular sovereignty ( Lorimer et al., 2026). On this basis, the case for classifying the ADR as populist rests not on its positions on immigration, gender, or national identity, but on its recurring portrayal of politics as a conflict between Luxembourgish citizens and unresponsive national or supranational elites, together with its demand that authority be returned to the people.

The ADR’s 2023 national election manifesto contains both elements. Party president Fred Keup argues that decisions are taken “over the heads of the people” and contrasts this with the ADR’s promise to return decision-making to Luxembourgish citizens. The manifesto connects this critique of governing elites to crime, COVID-19 restrictions, declining purchasing power, language loss, the weakening of the family, and demographic growth. Crucially, these claims are coupled with demands for referendums, direct democracy, and greater popular control over political decision-making. The ADR therefore goes beyond generic anti-establishment rhetoric by presenting itself as the vehicle through which the popular sovereignty can be restored. Because this people–elite opposition recurs across policy domains, its populism amounts to more than an isolated rhetorical device, even if it remains softer and less systematic than that of paradigmatic populist parties.

This vertical people-elite antagonism is accompanied by horizontal boundary-making ( Brubaker, 2020; Bennett, 2019; De Cleen, 2017; De Cleen & Stavrakakis, 2017). The ADR presents migrants and non-citizen residents seeking political rights as potential threats to Luxembourgish identity and political membership. This exclusionary dimension is nevertheless adapted to Luxembourg’s particular context. The party does not reject immigration outright, which would be politically and economically implausible in a country dependent on foreign and cross-border labour. Instead, it presents Luxembourg as a multicultural space rather than a multicultural nation: foreigners may be accepted as workers and residents without necessarily being incorporated into the symbolic and political core of the national community.

This helps explain Carls’ (2021) characterization of the party as practising “right-wing populism à la Luxembourgeoise.” Rather than primarily mobilizing economically marginalized groups, the ADR often appeals to comparatively privileged native Luxembourgers, particularly those employed in public administration and education, where Luxembourgish functions simultaneously as a marker of cultural belonging and a gatekeeping mechanism in the labour market.

The party’s ideological content should be assessed separately from its populism. From its manifesto, it is clear that the ADR combines national conservatism with classical liberalism, emphasizing national sovereignty, cultural preservation, direct democracy, and limited state interventions. It is Luxembourg’s most immigration-sceptical party ( Fetzer, 2011, p. 15), opposes voting rights for non-citizens, and favours more restrictive citizenship rules. It also adopts socially conservative positions on family, education, and gender, opposing gender-sensitive language, “gendered” schoolbooks, expanded transgender rights, and what it portrays as ideological “indoctrination” in schools, while taking more liberal positions on topics such as prostitution ( Carls, 2021, 2024).

These positions explain the right-wing and, in some respects, far-right affinities of the ADR’s discourse; they do not, by themselves, establish its populism. The party is therefore best understood as combining a right-wing, national-conservative host ideology with a recurring, though moderated, form of populism. This produces a triadic structure in which “the people” are opposed vertically to elites who disregard their sovereignty and horizontally to groups portrayed as lying outside, or threatening, the national political community ( Judis, 2016; Carls, 2024).

The ADR’s European affiliations have reinforced perceptions of a rightward shift. It joined the Alliance of Conservatives and Reformists in Europe in 2010 and became associated with the European Conservatives and Reformists (ECR) group after securing its first EP seat in 2024. These links have prompted accusations of far-right sympathies, which the party rejects. MEP Fernand Kartheiser has insisted that “there is no extreme right politically represented in Luxembourg. All these extremist tendencies are completely alien to our thinking.” Blau, however, argues that the party’s emphasis on national identity, Euroscepticism, moral decline, and immigration overlaps with central themes of the European far right ( RTL Today, 2023). De Jonge ( Journal.lu, 2023; RTL Today, 2024b) similarly identifies a rightward movement, particularly in the ADR’s growing focus on migration, gender, and climate, while stopping short of classifying it as extremist. The reported departure of former president and founding member Jean Mehlen over “ideological differences” further suggests a discernible ideological shift ( RTL Today, 2025a).

## Positions on ‘Europe’ and the Populism-Euroscepticism nexus

Luxembourg’s main parties, including the Christian Social People’s Party (CSV), the Democratic Party (DP), the Luxembourg Socialist Workers’ Party (LSAP), and the Greens (Déi Gréng), support EU integration. Euroscepticism therefore remains marginal. Other parties, including The Conservatives (right-wing), The Left (left-wing), and the Luxembourg Communist Party (left-wing), express varying forms of Euroscepticism, but the ADR is the most visible right-wing actor linking Euroscepticism to a recurring populist discourse.

The ADR’s position is best described as soft Euroscepticism or Eurocriticism. Its 2019 European election manifesto presented the party as “eurorealistic” or “euro-pragmatic” rather than anti-European, while its 2024 manifesto similarly frames the party as defending a strong Luxembourg within Europe rather than opposing European cooperation as such. This is consistent with Alexandra Schoos’ statement that, “Luxembourg needs Europe and the European Union ( Le Quotidien, 2024).” The ADR supports European cooperation insofar as it preserves peace, free movement, the internal market, and the common currency, but rejects excessive centralism, federalism, majority voting, and further transfers of sovereignty. Its preferred model is a “Europe of sovereign nations,” based on subsidiarity, national veto rights, and respect for national and cultural identities.

In EUPopLink terms ( Lorimer et al., 2026), the object of its Euroscepticism is both policy- and polity-related: it criticizes specific EU policies, including migration, climate, sanctions, enlargement, and defence integration, while also opposing the EU’s perceived trajectory toward deeper centralization. Its opposition therefore concerns the current practice and future direction of integration rather than the principle of European cooperation. In terms of nature, its critique is primarily sovereigntist and national-conservative, but becomes populist when EU decision-making is framed as removed from citizens, national parliaments, and democratic control.

The 2019 and 2024 manifestos both clarify why the ADR’s EU discourse is not merely conservative or sovereigntist. The party presents European integration as a process through which distant institutions, federalists, technocrats, and mainstream governing parties move power away from national peoples and member states. It calls for stronger parliamentary scrutiny, defence of the Luxembourg Compromise, unanimity in areas of vital national interest, and referendums on transfers of sovereignty, treaty changes, and major EU decisions. The populist element lies in this combination of anti-elite critique with an appeal to popular sovereignty and the will of national peoples.

At the European level, this vertical antagonism is again accompanied by horizontal boundary-making. The ADR supports legal migration and free movement within the internal market, which is particularly important in Luxembourg’s migration-dependent context. However, both manifestos defend stronger external borders, reject EU asylum quotas, criticize uncontrolled or illegal immigration, and insist that asylum and migration should remain national competences. The party presents EU migration policy since 2015 as a source of declining public trust in the EU and links migration control to sovereignty, security, and cultural identity. This approximates a triadic right-wing populist structure in which “the people” are positioned vertically against distant elites and horizontally against outsiders or uncontrolled migration from outside ( Judis, 2016; Carls, 2024).

Recent crises have reinforced rather than transformed the ADR’s orientation. During the Brexit debate, the ADR did not treat Brexit as a model for Luxembourgish withdrawal, but as evidence that federal Europe had failed to maintain democratic legitimacy, favouring a flexible “Europe à la carte” ( Högenauer, 2021). The 2019 and 2024 manifestos show continuity in this respect: both defend subsidiarity, unanimity in key areas, national sovereignty, and opposition to a federal Europe. The 2019 manifesto also prefigured later positions on Ukraine, Russia, and defence. It supported Ukraine economically and democratically, while warning against geopolitical escalation, defending unanimity in foreign policy, favouring pragmatic relations with Russia, and rejecting a European army. These positions help explain the party’s later criticism of EU sanctions, military escalation, and defence federalization after Russia’s invasion of Ukraine. The 2024 manifesto updates this position by condemning Russia’s invasion while maintaining a sovereignty-first and economically cautious approach to sanctions, defence, and foreign policy. ^ii^ Similarly, the ADR’s criticism of COVID-19 restrictions, opaque decision-making, and unilateral border closures reflected a longer-standing opposition to technocratic overreach and the transfer of national competences ( Lamour & Carls, 2022). The 2024 manifesto extends this critique to digital regulation and freedom of expression, presenting EU governance as technocratic, centralizing, and insufficiently responsive to citizens.

Taken together, the ADR’s Euroscepticism is soft but consistent. It accepts European cooperation while opposing federalization, majority voting, supranational competence expansion, and EU intervention in fields it defines as national. Its critique is not purely opportunistic: sovereignty, subsidiarity, national democracy, cultural identity, and democratic control are longstanding themes across both the 2019 and 2024 European election manifestos. Its salience nevertheless increases during crises and European elections, when EU institutions can be presented as distant elites overriding national peoples.

The ADR should therefore not be classified as populist simply because it advances immigration-sceptical, national-conservative, or socially conservative positions. These features locate it on the right and explain its partial overlap with the European far right ( De Cleen et al., 2018; Rooduijn & van Kessel, 2019). Its populism lies in the recurring construction of conflict between Luxembourgish or European peoples and unresponsive national or supranational elites, coupled with the claim that sovereignty must be restored through referendums, direct democratic control, and stronger national decision-making ( Canovan, 1999; Mudde, 2004; Taggart, 2000). It becomes specifically right-wing populist when this vertical people–elite logic is combined with horizontal boundary-making around migration, language, citizenship, and cultural sovereignty ( Judis, 2016; Carls, 2024). It can consequently be understood as a right-wing populist actor whose populism, although less systematic and confrontational than that of paradigmatic populist radical-right parties, is sufficiently recurrent to constitute more than occasional anti-establishment rhetoric ( van Kessel, 2014).

## Responses to Euroscepticism and consequences

Luxembourg’s pro-EU mainstream parties have responded to the ADR through adversarial, dismissive, and, more selectively, accommodative strategies ( Meguid, 2025). The dominant response is adversarial, particularly when mainstream actors perceive the ADR as crossing normative boundaries.

Adversarial responses are especially visible at European level, where the ADR’s alliances and positions can be linked to broader radical-right networks. Mainstream MEPs criticized its ECR affiliation: Charles Goerens (DP) described its allies as “not the best company,” while Marc Angel (LSAP) warned that this marked “the first time that Luxembourg has members on the benches of the extreme right or the populist right.” Tilly Metz (Déi Gréng) remarked that “it’s easy to run a campaign with populist slogans,” stressing the lack of substantive policy proposals. Christophe Hansen (CSV) characterized the ADR as part of “the far-right bloc” ( Luxembourg Times, 2024b).

The ADR’s position on Ukraine and Russia prompted similar reactions. Isabel Wiseler-Lima (CSV) commented, “We observe that the ADR has become more radicalized. They express understanding for various Russian positions, which we find deeply concerning” ( Virgule, 2024). Foreign Minister Xavier Bettel (DP) called Kartheiser’s Moscow visit “extremely problematic and unhelpful ( Luxembourg Times, 2025b).” Following Kartheiser’s expulsion from the ECR, Metz (Déi Gréng) responded with sharp irony, stating, “Too extreme even for the extremists! That’s quite an achievement ( RTL Today, 2025c).”

Language politics has produced similar reactions. Kartheiser’s insistence on using Luxembourgish in the EP was dismissed by Wiseler-Lima as a “circus act,” while Angel called it a populist “stunt”, arguing: “We conduct politics for our language, not with it. That is what the far-right and the populists might do ( RTL Today, 2024c).” Domestically, politicians across the ideological spectrum culture Minister Eric Thill (DP) likened the ADR’s narrative to “a juggler throwing a single ball” – an illusion of complexity without real substance. Similarly, MP Liz Braz (LSAP) warned that instrumentalising language promotes “subliminal xenophobia in political discourse ( RTL Today, 2024a).”

Social-media controversies have produced particularly forceful boundary-setting. In 2025, ADR MP Tom Weidig liked a post advocating the “extermination of LGBTQ” people, prompting a rare unanimous parliamentary resolution condemning all forms of hate speech ( RTL Today, 2025b). In 2019, a Facebook post by then-ADR politician Sylvie Mischel led Djuna Bernard (Déi Gréng) and Franz Fayot (LSAP) to compare the party with Germany’s right-wing populist Alternative for Germany (AfD), while Frank Engel (CSV) called on citizens to resist “sleeping fascism ( Reporter.lu, 2019).”

Such reactions defend democratic norms, particularly in response to hate speech, far-right associations, and pro-Russian positioning, but they may also serve a strategic partisan function. By portraying the ADR as extremist, irresponsible, or unserious, mainstream parties seek both to enforce normative boundaries and to restrict the legitimacy and electoral appeal of a right-wing competitor.

Dismissal is more common when the ADR’s EU criticism appears symbolic or electorally marginal. This strategy helps preserve the pro-European consensus, but it may also support the ADR’s claim that established parties ignore dissenting concerns. Limited accommodation occurs mainly on adjacent issues such as migration, public order, and national identity. De Jonge ( RTL Today, 2024b) notes that centrist politicians have occasionally echoed right-wing themes, as illustrated by controversies involving Simone Beissel (DP) on begging and Marc Lies (CSV) on refugees. These cases do not indicate that mainstream parties have become populist or Eurosceptic, but they show selective convergence on issues associated with the ADR’s agenda, potentially in response to electoral competition.

The consequences of the ADR’s Euroscepticism remain limited. It has not displaced Luxembourg’s pro-European consensus or generated support for withdrawal. EU opposition is also rarely its sole or principal electoral appeal; rather, it provides a sovereignty-based frame linking migration, language, public order, foreign policy, and democratic control. Its influence is therefore more visible in the increased salience and partial normalization of these themes than in a substantive reorientation of Luxembourg’s European policy.

## Conclusion

Luxembourg remains a least-likely case for hard Euroscepticism and populist radical-right-mobilization, but it is not insulated from these broader currents. The ADR combines a recurring people–elite antagonism and appeals to popular sovereignty with national-conservative and selectively nativist positions. Its acceptance of European cooperation and economically necessary immigration distinguishes it from more radical cases, while its boundary-making around language, citizenship, political membership, and national sovereignty gives its populism a specifically Luxembourgish form.

The ADR can therefore be understood as an example of ‘chameleonic populism’ ( Taggart, 2000, 2004): its discourse is responsive to local conditions and capable of challenging aspects of the political mainstream while continuing to operate within it. The Luxembourgish case shows that, in small, open, and deeply Europeanized states, right-wing populist Euroscepticism may be moderated rather than absent.

More broadly, the case underscores the need for context-sensitive approaches to the study of populism and Euroscepticism. It suggests that the populism-Euroscepticism nexus cannot be assessed solely through hard rejectionism, overt anti-EU mobilization, or explicit radicalism. Luxembourg broadens the analytical scope by demonstrating how populist and exclusionary claims can be adapted to strong pro-European norms, dependence on transnational labour, and deep structural integration within the EU. It thereby challenges analytical models derived primarily from larger states and highlights the flexible and context-dependent character of populist Euroscepticism.

## Ethics and consent

Ethical approval and consent were not required.

## Data Availability

No data are associated with this article.

## Footnotes

^i^
This country report traces the links between party-based populism and Euroscepticims in Luxembourg. The present country report was prepared as part of the EUPopLink COST Action ( Andreadis & Vasilopoulou, 2026) following the EUPopLink Theoretical Framework and guidelines ( Lorimer et al., 2026).

^ii^
Some of the ADR’s leading figures have, at times, openly expressed support for more radical right-wing positions. This tendency is particularly evident in the context of the war in Ukraine, where Kartheiser’s rhetoric has sparked controversy. His framing of the Ukraine war as a U.S.-Russia proxy conflict and criticism of EU alignment with the Biden administration echo narratives associated with Donald Trump (two-thirds of ADR voters thought that the election of Donald Trump is a good thing) ( Luxembourg Times, 2024a). Kartheiser’s 2025 trip to Moscow – and his remark that he would “rather fly to Moscow than talk to the Brussels bubble” – marked a clear opposition to the EU. The fallout was swift: on 4 June 2025, Kartheiser was expelled from the ECR group – the ADR continues to stand with Kartheiser ( Luxembourg Times, 2025a).
