# Supplementary material for: EUPopLink Country report - Luxembourg
Source: Open Res Eur. 2026 Aug 6;6:148. Originally published 2026 May 5. [Version 2] doi: 10.12688/openreseurope.23514.2 (PMC13560812; doi:10.12688/openreseurope.23514.2)
Supplement: Peer review history

# Reviewer report: Reviewer response for version 2

**DOI:** 10.21956/openreseurope.26646.r79232

**Published:** 2026-08-28

**Authors:** Robert Csehi

**Affiliations:** 1 Corvinus University of Budapest, Budapest, Hungary

Overall, I find this case study quite informative. If I understood the objective of the study correctly, it was to provide a descriptive account of the ADR and the current experience of Luxembourg with populism and Euroscepticism. In this sense, the study fulfilled its purpose. Having also read the previous reviews, I have nothing really substantial to add, I just want to highlight a few things that could be relevant for the future, nevertheless. Given that the study was carried out under the EUPoPLink cost action framework, I felt that the populist and Eurosceptic aspects were a bit too neatly separated from one another. In most cases, we already know a lot about the Eurosceptic AND the populist dynamics, and where we lack understanding is really about the interactions. I felt that the study could have done a better job addressing this. Instead of describing separately what populism AND Euroscepticism looks like under the ADR, this could really be integrated into a more theoretically driven research puzzle. In fact, the introduction starts with an interesting puzzle: given the special circumstances of Luxembourg, why do we see the emergence of both populist and Eurosceptic ideas at the party level? And once we see this, the natural question is WHY do we see a specific form of each, and how do they actually interact? Once again, I am not sure, what the general objective of the study was (beyond description), but this could be relevant for the future. This concern of mine was basically driven also by the fact that both concepts were a bit under-conceptualized. Now, I know that the conceptualization is supposed to appear in a central document, but it makes the study also a bit hard to evaluate this way. After all, a potential interaction between the two concepts should be based upon analytical linkages between the concepts' main building blocks. Why is the dyadic or triadic differentiation known in the populism literature relevant for Euroscepticism? Also, I feel that populism is not necessarily about the vertical or horizontal differentiation, because a lot of politicians do that. What differentiates populists from non-populists is really the moral division that they base this differentiation upon. What matter is not the people vs elite antagonism, but really the *corrupt *elite vs the *honest* people, or the *deserving* people vs the *undeserving *non-people. But, once again, we know this already, and applying this framework for a case study is informative, but I feel that the real work should start with the study of interactions between the two main concepts. 

 Overall, I found the piece informative, but theoretically less engaging, which - I admit - might not have been the objective of the study in the first place.

Is the case presented with sufficient detail to be useful for teaching or other practitioners?

Yes

Is the work clearly and accurately presented and does it cite the current literature?

Partly

If applicable, is the statistical analysis and its interpretation appropriate?

Not applicable

Are all the source data underlying the results available to ensure full reproducibility?

Yes

Are the conclusions drawn adequately supported by the results?

Yes

Is the background of the case’s history and progression described in sufficient detail?

Yes

Reviewer Expertise:

European integration, populism, Euroscepticism, East-Central Europe

I confirm that I have read this submission and believe that I have an appropriate level of expertise to confirm that it is of an acceptable scientific standard.

# Reviewer report: Reviewer response for version 1

**DOI:** 10.21956/openreseurope.25455.r73765

**Published:** 2026-06-03

**Authors:** Levan Kakhishvili

**Affiliations:** 1 ETH Zurich, Zürich, Zurich, Switzerland

I would like to thank the editorial team for giving me an opportunity to review a rather interesting case study: “EUPopLink Country Report – Luxembourg.” This manuscript offers a concise and engaging survey of populism and Euroscepticism in Luxembourg, centered on the Alternative Democratic Reform Party (ADR) and its place within a strongly pro-European political environment. The article’s main contribution lies in showing how a right-wing populist actor can adapt its message to the specific structural and socio-political conditions of a small, affluent, multilingual, and deeply EU-embedded state, producing a form of what the manuscript calls “chameleonic populism.” Overall, the manuscript is interesting, clearly written, and sufficiently detailed to give readers a solid overview of Luxembourgish political discourses on populism and Euroscepticism, and it should be indexed subject to minor revisions.

 The manuscript succeeds especially well in contextualizing Luxembourg as a country in which strong Euroscepticism has remained marginal despite social and economic features that, in other European contexts, often provide fertile ground for the radical right. The author effectively links this pattern to Luxembourg’s small size, economic reliance on European integration, dependence on foreign and cross-border labour, and the absence of organized anti-EU actors. The historical overview of the ADR is also clear and useful, tracing the party from its origins as a pension reform movement to its current role as the principal carrier of soft right-wing populist and Eurocritical discourse. The discussion of the ADR’s “populism from above,” especially its appeal to relatively privileged native Luxembourgers in public administration and education, is particularly insightful and one of the strongest parts of the manuscript.

 At the same time, the introduction would benefit from sharper conceptual framing. The manuscript uses terms such as populism, right-wing populism, extremism, and Euroscepticism in a generally intelligible way, but it would be stronger if it offered concise working definitions of these concepts near the outset. Because the article questions whether and how the ADR should be classified as right-wing populist, readers would gain from a clearer explanation of the criteria being used and of how those criteria relate to the Luxembourgish case. Such additions would also give the author an opportunity to cite relevant scholarly literature, which is prevalent on the topics of populism and Euroscepticism. This issue is especially relevant where the manuscript notes that some scholars classify the ADR as right-wing populist while others reject that label. Rather than only listing the two camps, the manuscript should briefly explain which definitions of right-wing populism they rely on and why these lead to different conclusions. That would provide a more nuanced understanding of the party’s ideological profile and help readers assess the classification debate more systematically.

 A few passages would also benefit from greater analytical nuance. On page 3, the statement that Eurobarometer figures indicate “a limited public demand for a strong Eurosceptic party” is plausible, but it may be somewhat too static. Public demand is not always pre-political or fixed; it can also be shaped, activated, or manufactured by political actors through issue framing, agenda setting, and strategic mobilization. A short acknowledgment of this possibility would strengthen the argument and avoid implying that existing public attitudes alone determine party political opportunity. Similarly, the claim that Luxembourg is frequently cited as a successful case of integration is interesting and relevant, but it would be more convincing if the manuscript specified more clearly who the immigrant population consists of. The national, socioeconomic, and legal composition of immigrants, i.e., whether they are mainly EU workers, non-EU migrants, refugees, asylum seekers, or highly skilled professionals, can significantly shape public perceptions and party strategies. Adding a sentence or two on this point would sharpen the empirical basis of the argument.

 There are also some language choices that merit reconsideration. The use of the expression “gender ideology” risks adopting a contested political label in the authorial voice, even if the intention is to describe ADR discourse. It would be preferable either to attribute the term explicitly to the party or to rephrase in more neutral analytical language. That would help preserve the manuscript’s critical distance and analytical precision.

 The section on mainstream party responses is informative and gives a convincing overview of how the ADR is challenged, criticized, and sometimes delegitimized by established political actors, particularly when it is perceived to cross normative or political red lines. The examples drawn from debates on European Parliament alliances, Ukraine, language politics, and social media controversies are well chosen and make the section vivid. At the same time, the analysis could go one step further by reflecting on whether these adversarial reactions are always fully warranted, or whether they may sometimes function as fear-mongering tactics aimed at discrediting a right-wing competitor that has the potential to gain social traction. Since the manuscript already notes that mainstream parties occasionally accommodate parts of the ADR agenda rhetorically, a slightly more reflective treatment of this tension between principled democratic containment and strategic partisan boundary-drawing would enrich the discussion.

 The manuscript’s conclusions are generally well supported by the evidence presented. The central claim that Luxembourg is not immune to populist and exclusionary politics, but that these politics take moderated and locally adapted forms in a small, prosperous, highly integrated state is persuasive and consistent with the empirical discussion. The article is also useful beyond the immediate case, because it encourages broader comparative reflection on how right-wing populism operates in contexts where overt anti-EU or anti-immigration radicalism is electorally constrained. For readers interested in teaching or applying the case comparatively, the manuscript already provides enough detail to be useful, though it would become even stronger with the conceptual clarifications noted above and a somewhat fuller description of Luxembourg’s migration profile.

 Overall, this is a thoughtful and worthwhile contribution that combines a clear overview of Luxembourg’s political landscape with a compelling argument about the contextual adaptation of populism and Euroscepticism. Its strongest elements are the discussion of the ADR’s ideological evolution, the treatment of language and national identity, and the broader framing of Luxembourg as a case of moderated or “chameleonic” right-wing populism. The revisions required are modest and mainly concern sharpening definitions, adding nuance to a few empirical claims, and slightly deepening the discussion of mainstream party responses and reproducibility. With these minor revisions, the manuscript would make a valuable contribution to the literature and would be suitable for indexing.

Is the case presented with sufficient detail to be useful for teaching or other practitioners?

Yes

Is the work clearly and accurately presented and does it cite the current literature?

Partly

If applicable, is the statistical analysis and its interpretation appropriate?

Not applicable

Are all the source data underlying the results available to ensure full reproducibility?

No source data required

Are the conclusions drawn adequately supported by the results?

Yes

Is the background of the case’s history and progression described in sufficient detail?

Yes

Reviewer Expertise:

Party competition, European Union

I confirm that I have read this submission and believe that I have an appropriate level of expertise to confirm that it is of an acceptable scientific standard.

# Response

**Published:** 2026-07-14

**Authors:** Lisa Verhasselt

Thank you for the generous and constructive review. I am grateful for the positive assessment of the report’s contribution. I have revised the report to address the conceptual and empirical points raised while preserving the concise country-report format required by the EUPopLink guidelines.

**Conceptual framing: **Thank you for this suggestion. The report was prepared as part of the EUPopLink COST Action and follows the shared EUPopLink Theoretical Framework and country-report guidelines. In order to keep the reports concise and comparable, the full definitions of the conceptual categories are provided in the EUPopLink guidelines and in Lorimer et al. (2026), rather than repeated extensively in each country report. However, I have revised the text to make clearer how the Luxembourg case relates to these categories, especially the distinction between populism, right-wing populism, Euroscepticism, conservatism, and the far right.

**Classification of the ADR:** I have revised the discussion of the ADR’s classification to avoid simply listing scholars who accept or reject the right-wing populist label. The revised text now explains why the classification is contested: a more restrictive approach may regard the ADR’s populism as too muted or intermittent, while a more context-sensitive approach identifies a strategically adapted form of “right-wing populism à la luxembourgeoise." The report now presents the ADR as a soft or borderline right-wing populist actor rather than as a straightforward populist radical right party.

**Criteria for identifying populism:** I have clarified that, under the EUPopLink definition, populism requires both anti-elite claims and appeals to popular sovereignty. The revised report therefore does not treat the ADR as populist simply because it is immigration-sceptical, socially conservative, or Eurosceptic. Instead, the classification is grounded in the party’s recurring portrayal of politics as a conflict between Luxembourgish citizens and unresponsive national or supranational elites, combined with its demand for referendums, direct democracy, and the restoration of popular control.

**Public demand and Eurobarometer figures:** Thank you for noting that the original wording risked presenting public demand as too static. I have revised this passage. The report now states that Luxembourg’s high levels of public support for the EU substantially narrow the electoral space for hard Euroscepticism, but that public demand is not fixed and may be activated or amplified by parties through agenda-setting and issue framing.

**Migration profile and integration:** I have expanded the introduction to provide a fuller description of Luxembourg’s migration and labour-market profile. The revised version specifies that Luxembourg has a high proportion of foreign residents and distinguishes between residents originating from neighbouring countries, other EU member states, and outside the EU. It also notes Luxembourg’s reliance on foreign and cross-border workers and its comparatively high number of asylum applications per capita. 

**Use of “gender ideology”: **Thank you for this important point. I have revised the relevant wording to avoid adopting contested party terminology in the authorial voice. The report now refers more neutrally to the ADR’s socially conservative positions on family, education, and gender, including its opposition to gender-sensitive language, “gendered” schoolbooks, expanded transgender rights, and what the party portrays as ideological “indoctrination” in schools.

**Mainstream party responses: **I have revised the section on mainstream-party responses by applying Meguid’s framework more explicitly. The report now distinguishes between adversarial, dismissive, and selectively accommodative responses. It also reflects more directly on the tension identified by the reviewer: adversarial reactions can function as principled democratic boundary-setting, especially in response to hate speech, far-right associations, or pro-Russian positioning, but they may also serve a strategic partisan function by limiting the legitimacy and electoral appeal of a right-wing competitor.

**Accommodation by mainstream parties:** The revised report also develops the point that mainstream parties occasionally accommodate adjacent parts of the ADR’s agenda. It now discusses selective convergence on issues such as migration, begging, public order, and national identity, while clarifying that these examples do not indicate that mainstream parties have become populist or Eurosceptic.

**Conclusion and comparative relevance: **Thank you for the positive assessment of the conclusion and comparative contribution. I have revised the conclusion to make this contribution more explicit and concise. The report now frames Luxembourg as a least-likely case showing that populist and Eurosceptic currents may emerge even under highly constraining conditions, but in moderated and locally adapted forms. It also clarifies the meaning of “chameleonic populism” as the ADR’s capacity to adapt right-wing populist discourse to Luxembourg’s pro-European, multilingual, migration-dependent, and consensus-oriented context.

**Reproducibility and scope:** In line with the EUPopLink country-report format, the revised report remains a concise secondary-research case study rather than an original statistical or manifesto-analysis article. I have nevertheless strengthened the empirical basis by drawing more explicitly on the ADR’s 2023 national election manifesto, and the 2019 and 2024 European election manifestos, alongside secondary literature and expert-informed classifications. No new figures, data, code, or author-list changes have been introduced.

**Overall revisions:** Overall, the revised version sharpens the conceptual framing, adds nuance to the discussion of public demand and migration, avoids contested terminology in the authorial voice, deepens the analysis of mainstream-party responses, and clarifies Luxembourg’s broader comparative significance. These revisions aim to address the reviewer’s main conceptual and empirical concerns while preserving the concise country-report format and remaining fully aligned with the EUPopLink guidelines.

# Reviewer report: Reviewer response for version 1

**DOI:** 10.21956/openreseurope.25455.r73769

**Published:** 2026-06-02

**Authors:** Chase Foster

**Affiliations:** 1 King's College London, London, England, UK

The article raises a number of interesting and potentially important points regarding the evolution of Luxembourg’s ADR and the broader dynamics of right-wing politics in Europe. The discussion of the party’s interactions with other right-wing parties in the European Parliament is especially insightful, particularly in showing how association with transnational far-right networks may contribute to ideological radicalization. At the same time, the article would benefit from a clearer conceptual framework and more sustained engagement with the literature on populism.

 A central issue concerns the distinction between “vertical” and “horizontal” exclusion. This distinction appears promising, but it is not sufficiently defined or developed. The concepts are introduced in the abstract and briefly referenced in the discussion of European Parliament debates, yet the article never clearly explains what differentiates the two forms of exclusion or how they relate to broader debates in the literature. The discussion recalls John Judis’ (2016) distinction between dyadic left-wing populism and triadic right-wing populism, where the latter involves a three-way relation between “the people,” “the elite,” and an out-group. However, this connection is never explicitly articulated. More importantly, the article never fully demonstrates that the ADR is in fact “triadic” in this sense, meaning that it engages simultaneously in both vertical exclusion (against elites) and horizontal exclusion (against outsiders or minorities).

 Relatedly, it is unclear whether the author intends to classify the ADR as a form of “chameleonic populism.” The term appears in the text, but the argument is not fully developed. Rather than simply citing other scholars who use this characterization, the article would benefit from making a more explicit and positive case for why the ADR should be understood in these terms. If this is indeed a central claim, it should be stated more directly and substantiated with clearer evidence.

 The article could also do more to explain why Luxembourg constitutes an important or theoretically interesting case. There seems to be a valuable opportunity here to frame Luxembourg as a least-likely case for right-wing populism. The emergence of a right-wing populist party in a wealthy, small, and historically moderate political system suggests that no country is entirely immune from these broader political developments, even if some countries are less likely to experience their most extreme manifestations. Framing the case in these terms would help strengthen the article’s broader comparative relevance.

 I would also encourage the author to provide more systematic evidence demonstrating that the ADR has become more right-wing over time. At present, many of the claims rely on impressionistic observations or isolated examples. It would strengthen the article considerably to incorporate more objective indicators or longitudinal evidence, whether through manifesto analysis, discourse analysis, expert surveys, or voting behavior.

 A related conceptual issue concerns the distinction between Euroscepticism, conservatism, the radical right, and populism. The article at times appears to conflate these categories. There are many mainstream and legitimate reasons for opposing further European integration, and Euroscepticism alone does not necessarily indicate a shift toward right-wing populism. Similarly, positions on issues such as constitutional sovereignty, public health mandates, the Luxembourgish language, or immigration may reflect conventional conservative politics rather than populism per se.

 For this reason, the article needs a much clearer conceptual discussion of what constitutes populism. It is not obvious from the evidence presented that the ADR is organized around the populist distinction between a virtuous people and a corrupt elite, which remains central to many influential definitions of populism (e.g. Mansbridge and Macedo 2019).

 Mansbridge, J. and Macedo, S., 2019. Populism and democratic theory. *Annual review of law and social science*, *15*(1), pp.59-77

 At several points, the ADR appears more like a conventional conservative or center-right party than a genuinely populist movement. The author notes that some scholars have described the ADR as “atypical” among populist parties (e.g. Carls 2021), yet the article does relatively little to convince the reader that the party should nevertheless be categorized as populist.

 Indeed, I found the discussion of the ADR’s interactions with right-wing populist parties in the European Parliament more persuasive than the domestic analysis itself. This section demonstrates how a relatively moderate conservative party may become radicalized through its connections with more extreme transnational networks. Yet even here, the evidence seems to point more toward movement in the direction of the far right than specifically toward populism.

 More broadly, the article would benefit from a clearer theoretical hook and a more explicit statement of its core contribution. At present, it is somewhat unclear whether the article is primarily descriptive or whether it is intervening in a broader theoretical debate. Clarifying the article’s central argument — particularly regarding how Luxembourg’s ADR should be conceptualized — would help sharpen both the analysis and the contribution.

 Finally, the article would benefit from deeper engagement with the literature on populism and related concepts. Terms such as populism, Euroscepticism, conservatism, and the radical right are overlapping but distinct categories, and the substantial literature on these topics could help the author refine the conceptual framework. The PopuList project, for example, may be particularly useful because it systematically distinguishes between populist, far-left, and far-right parties using expert-informed classification criteria.

 More generally, the literature review on populism feels somewhat underdeveloped. The following works would provide a strong foundation for clarifying the concept and distinguishing it from adjacent categories:

- Cas Mudde and Cristóbal Rovira Kaltwasser, *Populism: A Very Short Introduction*
- Jan-Werner Müller, *What Is Populism?*
- Ernesto Laclau, *The Populist Reason*
- Michael Kazin, *The Populist Persuasion: An American History*
- Margaret Canovan, *Populism*

Overall, the article contains a number of valuable observations and potentially important insights, particularly regarding the evolution of right-wing networks in Europe. However, the argument would be significantly strengthened by a clearer conceptual framework, more precise definitions, and a more explicit theoretical contribution

Is the case presented with sufficient detail to be useful for teaching or other practitioners?

Partly

Is the work clearly and accurately presented and does it cite the current literature?

Partly

If applicable, is the statistical analysis and its interpretation appropriate?

Not applicable

Are all the source data underlying the results available to ensure full reproducibility?

No source data required

Are the conclusions drawn adequately supported by the results?

Partly

Is the background of the case’s history and progression described in sufficient detail?

Partly

Reviewer Expertise:

comparative politics and comparative political economy

I confirm that I have read this submission and believe that I have an appropriate level of expertise to confirm that it is of an acceptable scientific standard, however I have significant reservations, as outlined above.

# Response

**Published:** 2026-07-14

**Authors:** Lisa Verhasselt

Thank you for the careful and constructive review. I have revised the report to sharpen the conceptual framing, better substantiate the classification of the ADR, and clarify the report’s comparative contribution, while remaining within the word limit and the structure required for EUPopLink country reports.

**Conceptual framework and engagement with populism literature: **Thank you for this suggestion. The report was prepared as part of the EUPopLink COST Action following the shared EUPopLink Theoretical Framework and country-report guidelines. In the interest of keeping country reports concise and comparable, the full definitions of conceptual categories are provided in the EUPopLink guidelines and in Lorimer et al. (2026), rather than repeated in detail in each country report. However, I have revised the text to connect the Luxembourg case more explicitly to the EUPopLink definition of populism and Euroscepticism. I also added references to relevant conceptual literature where space allowed.

**Vertical and horizontal exclusion:** Thank you for highlighting this. I have revised the discussion of vertical and horizontal exclusion to define the distinction more clearly. Vertical exclusion now refers to the ADR’s construction of conflict between “the people” and national or supranational elites, while horizontal exclusion refers to the construction of migrants, non-citizen residents, or progressive “ideologues” as threats to Luxembourgish or European identity. I have also linked this more explicitly to Judis’ triadic populism and clarified how the ADR combines both forms of exclusion.

**Triadic right-wing populism:** I have strengthened the argument that the ADR’s discourse approximates a triadic right-wing populist structure. The revised report now shows how the ADR positions “the people” vertically against national and EU elites, while also drawing horizontal boundaries against groups portrayed as lying outside or threatening the national political community. This is supported with evidence from the ADR’s 2023 national manifesto, and its 2019 and 2024 European election manifestos.

**Chameleonic populism:** Thank you for noting that this concept required further clarification. I have revised the report to state more explicitly why the ADR can be understood as an example of chameleonic populism. Rather than using the term only as a label, the revised version explains that it refers to the ADR’s capacity to adapt right-wing populist discourse to Luxembourg’s least-likely context: EU-critical without being anti-EU, culturally defensive without rejecting immigration as such, and anti-elite while often appealing to relatively privileged native Luxembourgers.

**Luxembourg as a least-likely case:** I have revised the introduction and conclusion to frame Luxembourg more explicitly as a least-likely case for hard Euroscepticism and radical right-wing populism. The report now emphasizes that Luxembourg’s wealth, small size, pro-European consensus, multiculturalism, and dependence on foreign and cross-border labour make hard anti-EU or uniformly anti-immigration politics unlikely to gain broad appeal. This strengthens the comparative relevance of the case.

**Evidence of the ADR’s rightward shift:** I agree that this point needed firmer empirical grounding. Within the limits of a concise country report, I have added more systematic evidence from the ADR’s 2023 national election manifesto, and the 2019 and 2024 European election manifestos, as well as from expert-informed classifications and secondary literature. The revised text now distinguishes between manifesto evidence, scholarly classification, and interpretive assessment. No original longitudinal manifesto or discourse analysis is introduced, as the country reports are designed as concise secondary-research case studies.

**Distinguishing Euroscepticism, conservatism, radical right politics, and populism**:

 I have revised the report to avoid conflating these categories. The text now states explicitly that the ADR should not be classified as populist simply because it is immigration-sceptical, socially conservative, national-conservative, or Eurosceptic. These elements help locate the party ideologically on the right and explain its overlap with far-right themes, but its populism lies in the recurring construction of a people–elite conflict and the claim that sovereignty should be restored through referendums, direct democratic control, and stronger national decision-making.

**What makes the ADR populist**: Thank you for raising this important point. The revised report now makes the case for classifying the ADR as a soft or contextually adapted populist actor more directly. It draws on the ADR’s 2023 manifesto, where party president Fred Keup argues that decisions are taken “over the heads of the people” and contrasts this with the party’s promise to return decision-making to Luxembourgish citizens. The report explains that this is not merely anti-establishment rhetoric because it is combined with appeals to popular sovereignty, referendums, and direct democratic control.

**Domestic evidence versus European affiliations:** I have revised the report so that the domestic analysis no longer relies mainly on the ADR’s European affiliations. The discussion of the party’s ECR links remains, but the classification of the ADR as populist is now grounded more clearly in domestic manifesto evidence and recurring claims about citizens, elites, referendums, sovereignty, language, and political membership. The European affiliations are treated as reinforcing perceptions of rightward movement, rather than as the sole basis for classification.

**Theoretical hook and core contribution:** I have sharpened the report’s core contribution. The revised version argues that Luxembourg demonstrates how populist Euroscepticism operates under constraining conditions. In a strongly pro-European, highly internationalized, and labour-dependent small state, hard rejectionism is unlikely to gain broad appeal; instead, right-wing populist and exclusionary claims appear in moderated, locally adapted forms. This argument is now stated more clearly in both the introduction and conclusion.

**Use of the PopuList and expert-informed classifications:** Thank you for this suggestion. I have revised the report to align the classification of the ADR more explicitly with the EUPopLink conceptual framework. Rather than relying on party labels alone, the revised version assesses the ADR in relation to EUPopLink’s definition of populism as combining anti-elite claims with appeals to popular sovereignty. The report also acknowledges the contested nature of the ADR’s classification in the secondary literature and expert-informed sources. It therefore presents the ADR not as a paradigmatic populist radical-right party, but as a soft and contextually adapted right-wing populist actor whose populism is recurrent, though moderated by Luxembourg’s pro-European and consensus-oriented political context.

**Scope of the literature review:** I agree that a fuller literature review would be valuable. However, the EUPopLink country reports are intended to provide concise empirical country accounts, not comprehensive theoretical literature reviews. The report is constrained by the project’s structure and 3,000-word limit. I have therefore not added an extended literature review, but I have strengthened the links to the EUPopLink framework and added selected conceptual references where they directly clarify the analysis.

**Overall revisions:** Overall, the revised report now more clearly distinguishes between Euroscepticism, conservatism, the radical right, and populism; provides manifesto-based evidence for the ADR’s populist discourse; explains vertical and horizontal exclusion more explicitly; substantiates the use of chameleonic populism; and frames Luxembourg as a theoretically relevant least-likely case. These revisions aim to address the reviewer’s main conceptual and empirical concerns while preserving the concise country-report format and remaining fully aligned with the EUPopLink guidelines.
